# *Cynanchum paniculatum* (Bunge) Kitag. ex H.Hara inhibits pancreatic cancer progression by inducing caspase-dependent apoptosis and suppressing TGF-β-mediated epithelial-mesenchymal transition

**DOI:** 10.3389/fphar.2024.1284371

**Published:** 2024-05-31

**Authors:** Chien-Shan Cheng, Yuan Wu, Jia-Bin Jin, Jia-Yue Xu, Pei-Wen Yang, Wen-Hua Zhu, Lan Zheng, Jing-Xian Chen

**Affiliations:** ^1^ Department of Traditional Chinese Medicine, Shanghai Jiao Tong University School of Medicine Affiliated Ruijin Hospital, Shanghai, China; ^2^ Department of Integrative Oncology, Fudan University Shanghai Cancer Center, Shanghai, China; ^3^ Department of Oncology, Shanghai Medical College, Fudan University, Shanghai, China; ^4^ Department of General Surgery, Pancreatic Disease Center, Ruijin Hospital, Shanghai Jiao Tong University School of Medicine, Shanghai, China; ^5^ Research Institute of Pancreatic Diseases, Shanghai Jiao Tong University School of Medicine, Shanghai, China

**Keywords:** *Cynanchum paniculatum* (Bunge) Kitag. ex H.Hara, apoptosis-induction, anti-invasion and anti-migration, caspase-dependent apoptosis, epithelial-mesenchymal transition

## Abstract

**Background:** *Cynanchum paniculatum* (Bunge) Kitag. ex H.Hara, a member of the Asclepiadaceae family, has a rich history as a traditional Chinese medicinal plant used to treat digestive disorders. However, its potential anti-cancer effects in pancreatic cancer remain largely unexplored.

**Aim:** This study delves into the intricate anti-pancreatic cancer mechanisms of *C. paniculatum* (Bunge) Kitag. ex H.Hara aqueous extract (CPAE) by elucidating its role in apoptosis induction and the inhibition of invasion and migration.

**Methods:** A comprehensive set of methodologies was employed to assess CPAE’s impact, including cell viability analyses using MTT and colony formation assays, flow cytometry for cell cycle distribution and apoptosis assessment, scratch-wound and Matrigel invasion assays for migration and invasion capabilities, and immunoblotting to measure the expression levels of key proteins involved in apoptosis and metastasis. Additionally, a murine xenograft model was established to investigate CPAE’s *in vivo* anti-cancer potential.

**Results:** CPAE exhibited time- and dose-dependent suppression of proliferation and colony formation in pancreatic cancer cells. Notably, CPAE induced apoptosis and G2/M phase arrest, effectively activating the caspase-dependent PARP pathway. At non-cytotoxic doses, CPAE significantly curtailed the metastatic abilities of pancreatic cells, effectively suppressing epithelial-mesenchymal transition (EMT) and downregulating the TGF-β1/Smad2/3 pathway. *In vivo* experiments underscored CPAE’s ability to inhibit tumor proliferation.

**Conclusion:** This study illuminates the multifaceted anti-proliferative, pro-apoptotic, anti-invasive, and anti-migratory effects of CPAE, both *in vitro* and *in vivo*. CPAE emerges as a promising herbal medicine for pancreatic cancer treatment, with its potential mediated through apoptosis induction via the caspase-dependent PARP pathway and MET suppression via the TGF-β1/Smad2/3 signaling pathway at non-cytotoxic doses. These findings advocate for further exploration of CPAE’s therapeutic potential in pancreatic cancer.

## 1 Introduction

Pancreatic cancer is a devastating malignancy within the digestive system (Siegel et al., 2019; Cai et al., 2021), characterized by a dire 5-year survival rate of approximately 8%–9% (Collaborators, 2019; Maisonneuve, 2019). Late diagnosis and atypical onset contribute to its dismal prognosis (Collaborators, 2019; Maisonneuve, 2019). Current treatment modalities, encompassing surgical resection, radiotherapy, chemotherapy, and other systemic therapies (Neoptolemos et al., 2018), face significant limitations, often leading to recurrence and metastasis. Consequently, there is an urgent need to unearth novel therapeutic approaches for this formidable disease.

Complementary and alternative medicine, particularly Chinese Medicine (CM), has garnered substantial attention among cancer patients (Yang et al., 2015), widely accepted for its efficacy in both treatment and prevention (Jiang et al., 2019; Gao et al., 2021; Wu et al., 2021). Chinese herbal medicine (CHM) boasts a rich history of clinical practice and literature, offering a wellspring of potential botanical anti-cancer agents. While ancient CM literature lacks a specific cancer concept, it frequently references cancer-like symptoms, making it relevant to contemporary anti-cancer research (Tang et al., 2009). Therefore, identifying CHM with traditional symptomatic use of cancer-related symptoms and with potential and ethnopharmacological relevance to its anti-cancer activities.

The dry roots and rhizomes of *Cynanchum paniculatum* (Bunge) Kitag. ex H.Hara (*Xuchangqing* in Chinese), (herb’s name has been checked with http://theplantlist.org), a species of Asclepiadaceae plants, were first recorded in Shennong’s Materia Medica Classic, an ancient pharmacopeia in China (Han Dynasty, 206 BC to 220 AD) as the top-grade drug (tonic and non-toxic). Ethnopharmacologically, according to the 2015 edition Chinese Pharmacopoeia, *C. paniculatum* (Bunge) Kitag. ex H.Hara is frequently used in herbal decoction in aqueous extract form (the CPAE) to alleviate various aliments, including resolve masses, pain relief, and detoxification (Chinese Pharmacopoeia Commission, 2015; Chen et al., 2020; Zhou et al., 2020). Recent clinical trials have validated its effectiveness in pancreatitis treatment, which is closely linked to pancreatic cancer risk. (Zheng and Gao, 2008).

In the Chinese medicine theory, a tumor is seen as a combination of dampness and toxicity, which are also key characteristics of inflammation (Tang et al., 2009), a recognized hallmark of cancer (Colotta et al., 2009). This connection suggests that the potential anti-cancer and anti-inflammation effects of CPAE may be explained by its ability to eliminate wind-damp or toxicity under CM theory. Moreover, inflammation can lead to the production of cytokines that promote tumor progression. A clinical study observed significant reductions in plasma endotoxin, IL-6, and TNF-α levels in patients with pancreatitis after receiving *xuchangqing* decoction treatment (Zheng, Y., 2008), indicating that CPAE might inhibit inflammation-triggered tumorigenesis. Another clinical study demonstrated the effectiveness of *Aikang* Decoction, with CPAE as a prominent component, in treating pancreatic cancer pain (Pan, 2017). However, the precise mechanism through which it combats pancreatic cancer remains to be fully investigated.

Modern pharmacological studies reveal that CPAE is rich in phenolic compounds, as illustrated by a high-performance liquid chromatography (HPLC) study by Weon, et al. on *C. paniculatum* (Bunge) Kitag. ex H.Hara (Weon et al., 2012). The identified compounds, such as paeonol, CPB64, and CPB54, have been found to possess anti-inflammatory, anti-allergic, anti-cancer, and immunomodulatory effects (Lee et al., 2003; Min et al., 2010; Kim et al., 2012; Liu, 2016; Cheng et al., 2020; Zhou et al., 2020). Paeonol, a major CPAE component, constitutes about 1% of the total dry *C. paniculatum* and is also a major active ingredient in *xuchangqing*. Paeonol had been extensively studied for its anti-proliferative effect via arresting cell-cycle at S-phase, inducing apoptosis via PI3K/AKT pathway in gastric, colorectal and hepatic cancer (Ji et al., 2007; Chunhu et al., 2008; Li et al., 2010). It functions by inhibiting tumor metastasis through the disruption of proinflammatory cytokine-mediated NF-κB/STAT3 pathways, TGF-β1/Smad signaling, and epithelial-mesenchymal-transition (Zhang et al., 2015; Cheng et al., 2020). Although *xuchangqing* is frequently applied in CHM for cancer patients, there is still a need for a more comprehensive exploration of its anti-tumor effects and the underlying mechanisms.

## 2 Materials and methods

### 2.1 Chemicals and reagents

CPAE (Cat. No. 20071561) was acquired from Jiangyin Tianjiang Pharmaceutical Co., Ltd. (Jiangsu, China) and its authenticity was confirmed by Prof. Lan Zheng at Ruijin hospital, Shanghai, China. A sample (No. 2021CP02) was deposited in the Department of Traditional Chinese Medicine of Shanghai Ruijin Hospital.

To prepare the CPAE extract, initially, CPAE particles were immersed in phosphate buffer saline (PBS, 8121467, Gibco, United States) at a concentration ten times that of the CPAE dose for 30 min. Subsequently, they were boiled at 100°C for 150 min. The resulting decoction was then filtered using 0.22 μM syringe-driven filters (Cat. No. 211024-052-A, JET BIOFIL, China). Next, the filtered decoction was boiled once more in PBS, this time at a concentration five times that of the CPAE dose) and at a temperature of 100°C for 75 min. After this, the decoction was filtered again with 0.22 μM syringe-driven filters. The two filtrates were combined and concentrated under vacuum conditions (vacuum degree >0.08 MPa; temperature 60°C) to obtain an extract. Finally, this extract was diluted separately with two types of serum-free medium: Dulbecco’s Modified Eagle’s Medium (DMEM, Cat. No. D211010, BasalMedia, China) and Iscove’s Modified Dulbecco’s Medium (IMDM, Cat. No. 10-016-CVR, Corning, United States) until it reached a final concentration of 1.2 mg/mL. TGF-β1 was diluted to a concentration of 100 μg/mL (Cat. No. 100-21, PeproTech, United States). All of these preparations were made in readiness for the subsequent procedures.

### 2.2 Cell line and cell culture

We obtained Panc-1 and Capan-1 cell lines, which are human pancreatic cancer cells, from American Type Culture Collection (ATCC). Panc02 murine pancreatic cancer cell (C56BL/6 origin) was obtained from the Frederick National Laboratory for Cancer Research (Frederick, MD, United States). Panc-1 and Panc02 cells were maintained in DMEM supplemented with 10% fetal bovine serum (FBS, Cat. No. 2053264, B.I., China), and 1% penicillin and streptomycin (Cat. No. 2321126, Gibco, United States). Capan-1 cells, on the other hand, were cultured in IMDM with 20% FBS and 1% penicillin and streptomycin. All cells were cultured in a sterile incubator at 37°C with 5% CO_2_.

### 2.3 Cell viability assay

In the cell viability assay, approximately 5,000 cells were initially seeded in 96-well plates, with each well containing 100 μL of the medium. These cells were allowed to incubate overnight. Subsequently, a range of CPAE concentrations, varying from 0 to 400 μg/mL, was applied to the cells for incubation periods of 24, 48, and 72 h. Following the respective incubation times, 3- (4,5)-dimethylthiahiazo (-z-y1)-2,5-di-phenytetrazoliumromide (MTT, 5 mg/mL, Sigma-Aldrich, United States) was introduced to the cells. After a 4 h incubation period with MTT, the culture medium was meticulously aspirated from each well, and 200 μL of dimethyl sulfoxide (DMSO, Sigma) was added to dissolve the formazan crystals. Subsequently, the optical density at 570 (OD570) was measured using a microplate reader (BioTek Instruments, Vermont, United States). For subsequent apoptosis experiments, CPAE was used at concentrations of 5, 10, and 20 μg/mL. In contrast, relatively lower concentrations of CPAE (1.25 and 2.5 μg/mL) were employed in invasion and migration experiments.

### 2.4 Colony formation assay

For the colony formation assay, Panc-1 and Capan-1 cells were cultured in 6-well plates at a density of approximately 1,000 cells per well. Fresh culture medium containing either 0, 10 and 20 μg/mL of CPAE that are lower than the determined IC_50_ concentration for both cell lines was added to the wells after overnight incubation. The cells were then allowed to incubated for a period of 10 days. After this incubation period, the colonies that had formed were fixed with 4% paraformaldehyde (PFA, P0099, Beyotime, China) for 2 h. Subsequently, they were stained with crystal violet (C0121, Beyotime, China) for 15 min. Following the staining, the culture plates were rinsed under running water. To capture images, a white paper was placed beneath the 6-well plate, and photographs were taken in a well-lit environment. This experiment was conducted in triplicate for reliability.

### 2.5 Apoptosis assay

For the apoptosis assay, cells were cultured in 6-well plates at a density of 1 × 10^5^ cells per well. The existing medium was replaced with fresh medium containing various concentrations of CPAE (5, 10, and 20 μg/mL, all of which are under the obtained IC_50_ concentration) and incubated for 48 h. Following this incubation period, the cells were harvested after trypsinization using EDTA-free trypsin (No. C0205, Beyotime, China) and centrifuged at 300 *g* for 5 min at 4°C. The collected cells were then washed twice with pre-cooled PBS by centrifugation at 300 *g* and 4°C for 5 min. The cells were resuspended in Binding Buffer (40302-C, YEASEN, China), and subsequently, Annexin V-FITC (40302-A, YEASEN, China) and PI staining solution (Propidium Iodide, 40302-B, YEASEN, China) were added to cell suspension. The cells were shielded from light and allowed to reacted at room temperature for 15 min. Within 1 h, the samples were analyzed using a flow cytometer (CytoFLEX, Beckman-Coulter, United States) in accordance with the manufacturer’s instructions. The gating strategy used for flow cytometry analysis was performed as per the manufacturer’s instructions. Flowjo software (version 10.8.1) was utilized for data analysis. This experiment was conducted in triplicate for reliability.

### 2.6 Cell-cycle analysis

Cells were seeded at a density of 1 × 10^5^ per well in 6-well plates. Following an overnight incubation, the cells were treated with CPAE (5, 10, and 20 μg/mL) for 48 h. Both adherent and floating cells were subsequently collected. These cells were fixed with 70% ethanol overnight at 4°C. Meanwhile, PI (Propidium Iodide, 40301-B, YEASEN, China) and 10 μL RNase A solution (40301-A, YEASEN, China) were mixed with staining buffer (40301-C, YEASEN, China) for the future use. This prepared PI staining solution was then added to each cell sample to resuspend the cells. The cell suspension was incubated at 37°C in the dark for 30 min before analysis using flow cytometry (CytoFLEX, Beckman-Coulter, United States). The gating strategy employed for flow cytometry analysis followed the manufacturer’s instructions, and data analysis was performed using Flowjo software (version 10.8.1). This experiment was repeated three times to ensure accuracy and consistency.

### 2.7 Scratch-wound assay

Cells were cultured in 6-well plates until they reached approximate confluence. To create a scratch wound, the near confluent cells were gently scraped perpendicular to the bottom of the plate. Fresh culture medium containing CPAE at concentrations of 1.25, and 2.5 μg/mL that are lower than IC_10_ concentration (considered non-cytotoxic) for both cell lines at 48 h was then added to the cells. The rate of wound closure was monitored by capturing microscope images at 0, 24, and 48 h (Axio Scope. A1, Zeiss, Germany). For data analysis, ImageJ (National Institutes of Health, United States) and GraphPad Prism 9 were used to perform statistical calculations. This experiment was conducted three times to ensure the consistency and reliability of the results.

### 2.8 Matrigel invasion assay

To prepare the chamber for the invasion assay, a mixture of Matrigel (Corning, United States) and serum-free medium was created in a 1:5 ratio. This mixture was then placed in the upper chamber of a transwell chamber (8.0 μM pore size, Corning, United States). The coated chamber was incubated at 37°C for 2 h. Subsequently, approximately 2 × 10^4^ cells were added to the upper chamber and allowed to incubate overnight. The culture medium was then replaced with fresh medium containing varing concentrations of CPAE at 1.25, and 2.5 μg/mL. After 48 h, any remaining cells on the upper surface of the chamber were carefully removed using a cotton swab. To fix the cells that had invaded through the chamber, 4% PFA (P0099, Beyotime, China) was applied for a 2-h period. Subsequently, the cells were stained with crystal violet (C0121, Beyotime, China). The cells that had successfully invaded through the chamber were observed and quantified using an optical microscope (OLYMPUS, Japan). For data analysis, ImageJ (National Institutes of Health, United States) and GraphPad Prism 9 were used to perform statistical calculations. This experiment was conducted three times to ensure the consistency and reliability of the results.

### 2.9 Immunoblotting assay

Panc-1 and Capan-1 cells were incubated with the desired concentration of CPAE for 48 h. Total protein was then extracted with RIPA (P0013B, Beyotime, China), which contained protease and phosphatase inhibitors (P1045, Beyotime, China). Extracted proteins were lysed on ice. Afterward, the protein was centrifuged at 12,000 rpm for 15 min at 4°C. And then, the Bradford protein kit (Cat No. P0006, Beyotime, China) was used to quantify the protein concentrations. The scale of the gel was selected according to the molecular weight of the protein, which was then electrophoresed and transferred to a membrane. The membrane was blocked with 5% bovine serum albumin (BSA, B92947, ABCONE, China) for 2 h. Primary antibodies Bax (#5023), Bcl-2 (#3498), Caspase3 (#9662), Caspase9 (#9504), Cleaved-PARP (#5625), E-cadherin (#3195), N-cadherin (#13116), Smad2 (#5339), Smad3 (#9523), p-Smad2 (#18338), p-Smad3 (#9520), Vimentin (#5741), and GAPDH (#5174) were added overnight at 4°C. Then, anti-rabbit IgG, HPR-linked antibody (#7074, CST, United States of America) was added. Membranes were completely immersed in the super ECL detection reagent (36208ES60, Yeasen, China), protected from light for 3 min at room temperature, and exposed in a Tanon 6100 chemiluminescence imaging system (Tanon, Shanghai, China). Finally, ImageJ (National Institutes of Health, United States of America) and GraphPad Prism 9 were used for quantitative analysis.

### 2.10 *In-vivo* studies

Four-to six-week-old male C57BL/6 J mice were purchased from Shanghai Jihui Laboratory Animal Breeding Co., Ltd. (animal certificate no. 20170012021693) on 18 February 2022. The experimental animal protocol was approved by the Experimental Animal Ethics Committee of Fudan University Shanghai Cancer Center (FUSCC-IACUC-S2022-0397). We guaranteed that animal experiments comply with the 3R principles and did our best to strive for animal welfare.

#### 2.10.1 Subcutaneous pancreatic tumor-bearing mouse model

1×10^7^ panc02 pancreatic cancer cells were harvested and suspended in 1 mL PBS (8121467, Gibco, United States), and 200 μL of cells were slowly seeded into the right axilla. One week after tumor inoculation, when tumors reached a volume of approximately 10 mm^3^, tumor-bearing mice were randomly divided into CPAE-Low group (0.75 g/kg/2d, *n* = 3) and CPAE-High group (1.5 g/kg/2d, *n* = 3). The vehicle control group (*n* = 3) was given an equal volume of PBS (0.2 mL/time). The dosage of CPAE is calculated based on the commonly used dosage in traditional Chinese medicine clinics and converted for human-to-mouse translation. This conversion is performed to ensure the feasibility and reliability of our experiments in the context of potential clinical applications. Mice were weighed weekly and tumor length and width are measured weekly. After 4 weeks of CPAE treatment, CO_2_ suffocation was used to euthanize mice. Tumors were excised and measured.

#### 2.10.2 Histology and immunohistochemistry analyses

First, the subcutaneous tumor, heart, liver and kidney, tissue were paraffin-embedded (BMJ-IB, Tianjin Tianli Aviation Electromechanical, China). Paraffin slices were baked at 62°C for 1 h (DHG-9960A, Shanghai Sanfa Scientific Instruments, China). The slices were then immersed in xylene (10023418, Sinopharm Chemical Reagent Co., Ltd., China). Then, the slices were immersed in absolute ethanol, 95% ethanol, and 70% ethanol (100092683, Sinopharm Chemical Reagent Co., Ltd., China) for 1 min. After washing in distilled water for 5 min, hydrogen peroxide (73113760, Sinopharm Chemical Reagent Co., Ltd., China) was added 100 μL per slide and then slides were incubated for 10 min. Further, the washed slides were placed in a boiling antigen repair solution (1 mmol Tris-EDTA pH = 9.0) (E9884, Sigma, United States) for 15 min and rinsed. Following, 5% BSA blocking solution (B2064, Sigma, United States) was added to each slice, which were incubated for 20 min at room temperature. Subsequently, the diluted anti-Bax (1:200, T40051S, Abmast), anti-Bcl-2 (1:200, 15071S, CST), E-cadherin (1:300, 3195S, CST), anti-Vimentin (1:400, 5741S, CST), anti-TGF-β (1:200, ab215715, Abcam), anti-p-Smad2/3 (1:100, 8828S, CST) were added to slides at 4°C overnight. Then the secondary antibodies (K5007, DAKO, China) were added to the washed slices the next day and the slides were incubated at 37 °C for 30 min. After washing again, DAB was added to each slice to develop color. Slices were then counterstained with hematoxylin (Bry-0001-01, Runnerbio, China) for 30 s. Dehydration of 70%, 95%, 95%, 100% ethanol (100092683, Sinopharm Chemical Reagent Co., Ltd., China) was performed. Finally, slides were sealed with neutral sealant (10004160, Sinopharm Chemical Reagent Co., Ltd., China). A microscope visualized the results (Nikon Eclipse Ci-L, Japan).

### 2.11 Statistical analysis

GraphPad Prism 9 was used for statistical analysis. The data was expressed in Mean ± SD. The two sets of data were compared according to the normal distribution and the homogeneity of the variance, and the non-paired *t*-test was used. One-way ANOVA was used to compare data between multiple sets. The level of significance was set at *p* < 0.05, *p* < 0.01, *p* < 0.001.

## 3 Results

### 3.1 CPAE inhibits proliferation of pancreatic cancer cells *in vitro*

**FIGURE 1:**
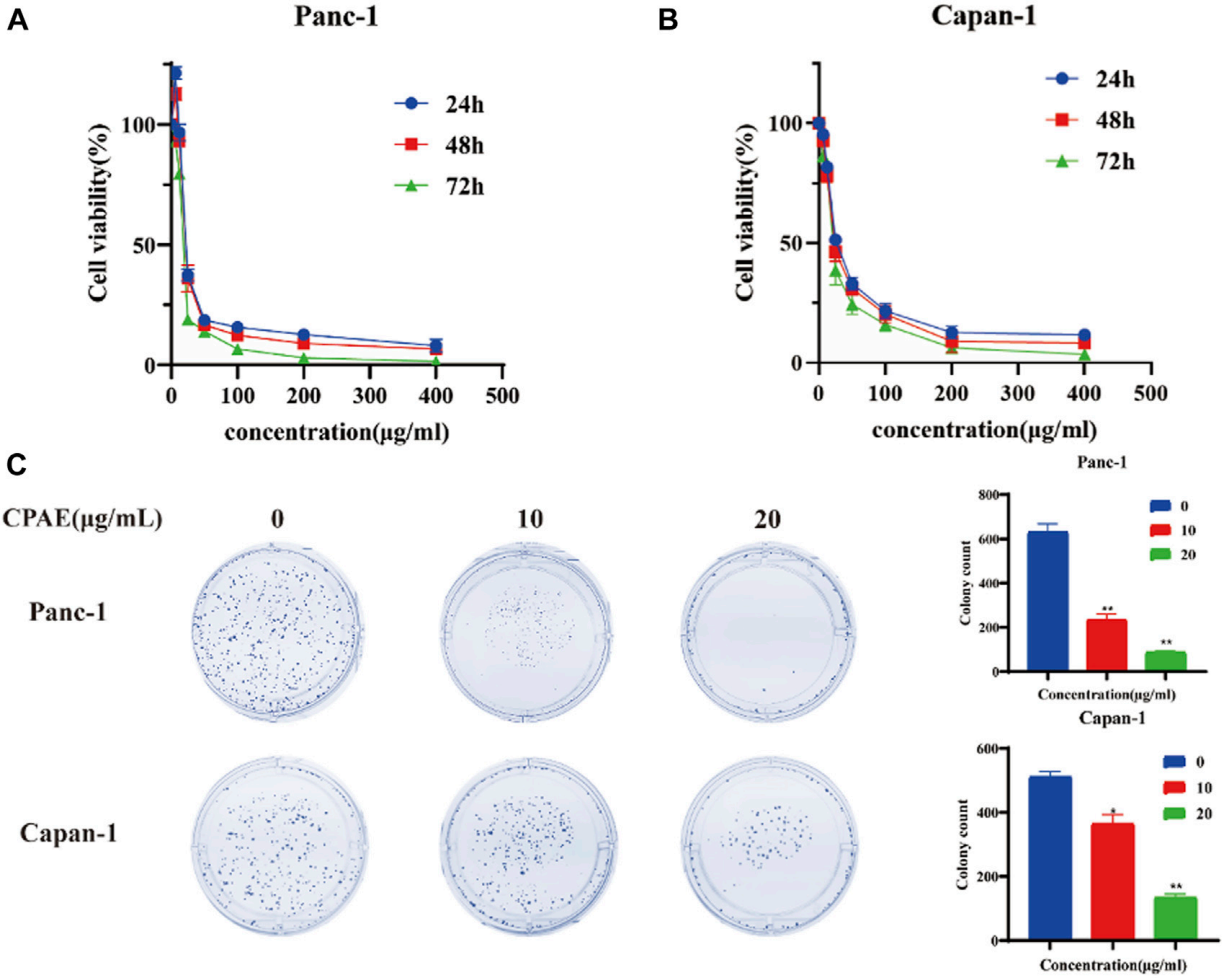
Antiproliferative effect of CPAE on pancreatic cancer cells. **(A)** In Panc-1 cells, cell viability at 24, 48 and 72 h after CPAE interventions at doses of 0, 6.25, 12.5, 25, 50, 100, 200, 400 μg/mL **(B)** In Capan-1 cells, cell viability at 24, 48 and 72 h after CPAE interventions at doses of 0, 6.25, 12.5, 25, 50, 100, 200, 400 μg/mL; **(C)** CPAE (10, 20 μg/mL) reduces colony formation of pancreatic cancer cells. And histograms show that CPAE (10, 20 μg/mL) inhibits clonal formation of Panc-1 and Capan-1 cells. *p < 0.05, **p < 0.01.

To assess the impact of CPAE on pancreatic cancer cell proliferation, we conducted the MTT assay. Upon treating the cells with various concentrations of CPAE, we observed that CPAE effectively suppressed the proliferation of pancreatic cancer cells in a time- and dose-dependent manner (Figures 1A, B, additional data below a concentration of 50 μg/mL can be found in Supplementary Figure S1). A 50% inhibitory concentration (IC_50_) of CPAE on cells was determined after 48 h. The IC_50_ of Panc-1 cells was 20.56 μg/mL, and that of Capan-1 cells was 27.33 μg/mL. At the concentration of 25 μg/mL, the growth inhibition rate were 34.9% ± 2.4%, 36.4% ± 5.3%, and 19.4% ± 0.9% at 24, 48, and 72 h, respectively for Panc-1 cell, while that of the Capan-1 cells were 51.6% ± 1.6%, 46.7% ± 3.6%, and 38.9% ± 6.1%, respectively. The results demonstrated that CPAE time-dependently suppressed pancreatic cancer cell proliferation. Concentrations, 10 μg/mL and 20 μg/mL that are lower than that of the IC_50_ were selected for colony formation assay. The results showed that after 12 days, different concentrations of CPAE could reduce the colony formation of the pancreatic cells (Figure 1C).

### 3.2 CPAE induces apoptosis and cell cycle arrest in pancreatic cancer cells *in vitro*

**FIGURE 2:**
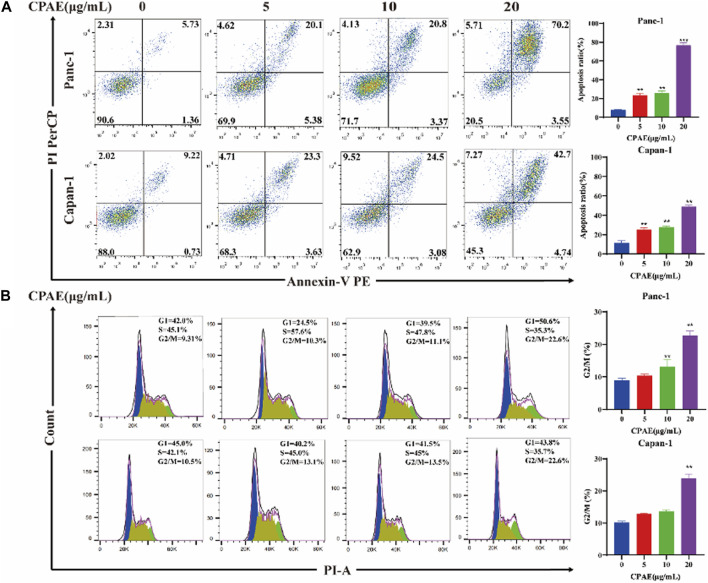
Apoptosis and cell cycle detection of pancreatic cancer cells treated by CPAE. **(A)** CPAE (0, 5, 10, 20 μg/mL) increases the apoptotic ratio of Panc-1 and Capan-1 cells. **(B)** 5, 10, and 20 μg/mL of CPAE increases the proportion of the G2/M phase of Panc-1 and Capan-1 cells. *p < 0.05, **p < 0.01, ***p < 0.001.

Apoptosis of pancreatic cancer cells was assessed through flow cytometry analysis. The results showed that following a 48-h incubation with CPAE at concentrations of 5, 10, and 20 μg/mL, the apoptosis ratio of the Panc-1 cells increased, which was 25.48% ± 2.14%, 26.17% ± 2.27%, and 73.75% ± 2.88%, respectively. The apoptotic proportions in Capan-1 cells also increased after 48 h of incubation with CPAE at 5, 10, and 20 μg/mL, which were 25.27% ± 1.82%, 27.58% ± 1.31%, and 47.44% ± 1.46%, respectively (Figure 2A). Furthermore, cell cycle analysis indicated that following a 48-h incubation with CPAE at concentrations of 5, 10, and 20 μg/mL, the proportion of Panc-1 cells in the G2/M phase displayed a significant increase, measuring at 10.3%, 11.1%, and 22.6%, respectively. Similarly, the proportion of Capan-1 cells at the G2/M phase also increased after 48 h of incubation with CPAE at 5, 10, and 20 μg/mL, which was 13.1%, 13.5%, and 22.6%, respectively (Figure 2B). The results showed that CPAE induces apoptosis by arresting pancreatic cancer cells in the G2/M phase of the cell cycle.

### 3.3 CPAE induces apoptosis through the caspase-dependent PARP pathway *in vitro*

**FIGURE 3:**
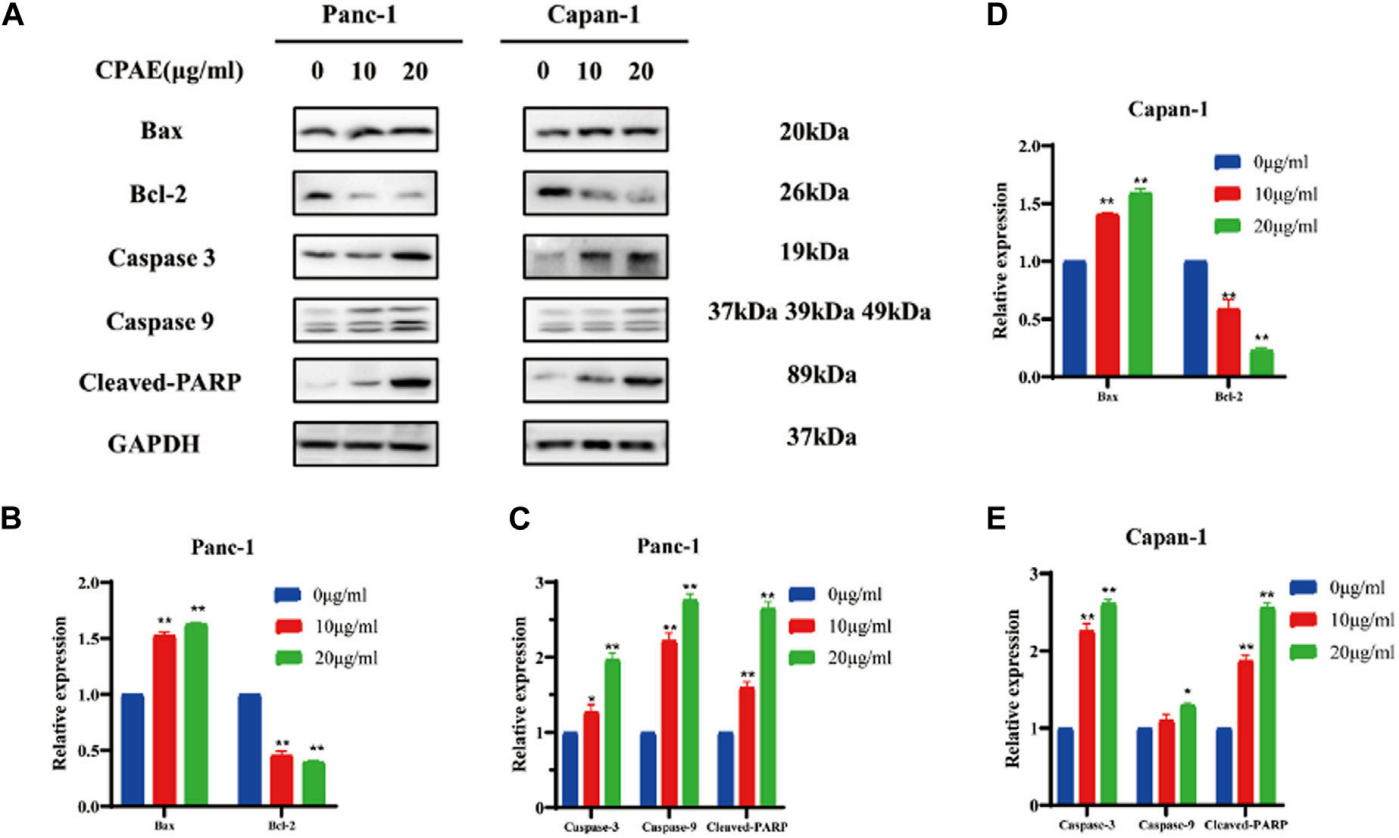
CPAE promotes apoptosis of pancreatic cancer cells through the caspase-dependent PARP pathway. **(A)** Protein levels of Bax, Bcl-2, caspase-3, caspase-9, and cleaved-PARP in cells after CPAE treatment for 48 h by immunoblotting assay **(** **B–E** **)** Histogram shows the levels of proteins above in Panc-1 and Capan-1 cells. *p < 0.05, **p < 0.01.

Bcl-2 and Bax act in the form of dimers *in vivo*, and the ratio between these two proteins plays a crucial role in determining whether a cell survives or undergoes apoptosis. We examined the expression of apoptosis-related proteins after 48 h treatment with varying concentrations of CPAE (Figure 3A). Following CPAE treatment, the levels of Bax protein increased within the cells, while the levels of Bcl-2 decreased. These changes suggested that CPAE has the ability to induce apoptosis in pancreatic cancer cells. In addition, caspase-9 is known to cleave caspase-3, initiating the activation of the caspase cascade (Choudhary et al., 2015). PARP, essential for cell stability and survival, could be cleaved by various caspases, leading to the inactivation of its enzymatic activity and ultimately contributing to cellular instability (Wang et al., 2019; Slade, 2020). We observed that after treatment with CPAE, the levels of caspase-3, caspase-9, and cleaved-PARP increased (Figures 3B–E). These findings indicates that CPAE promotes apoptosis via the caspase-dependent PARP pathway *in vitro*.

### 3.4 Non-cytotoxic doses of CPAE inhibit pancreatic cancer cell migration and invasion

**FIGURE 4:**
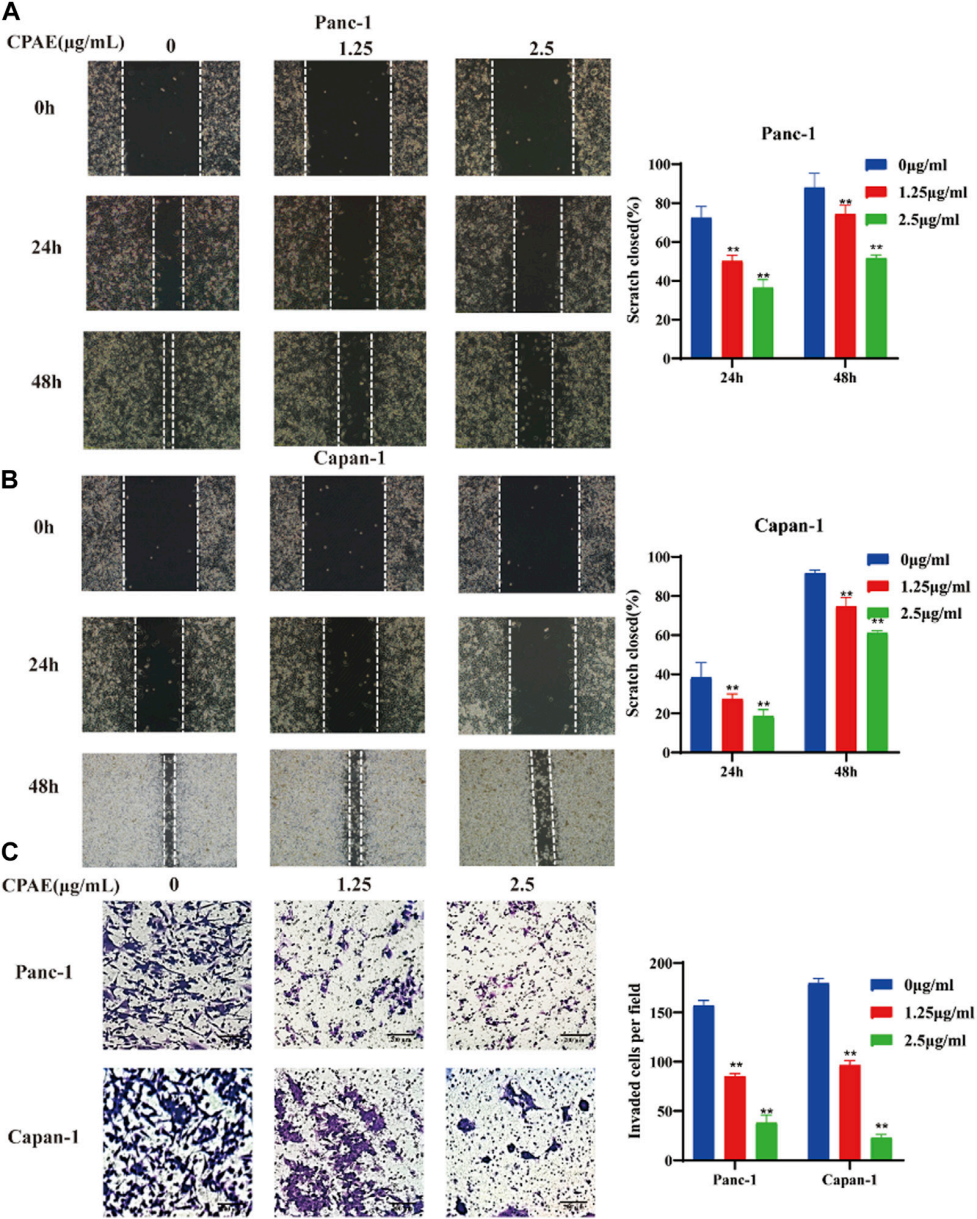
CPAE reduces the metastasis of pancreatic cancer cells. **(A,B)** After treating cells with 1.25 or 2.5 μg/mL CPAE, the migration of the cells is determined with a scratch wound healing assay. Statistical comparison of scratch healing after CPAE treatment in Panc-1 and Capan-1 cells. **(C)** Boyden Chamber assay demonstrates that non-cytotoxic CPAE reduces the invasion of pancreatic cancer cells. Statistical comparison of invaded cells after CPAE treatment. **p < 0.01.

To assess the impact of non-cytotoxic doses of CPAE on the anti-invasion and anti-migration capabilities, we treated pancreatic cancer cells with CPAE at the concentration of 1.25 μg/mL and 2.5 μg/mL for 0, 24, and 48 h. The scratch-wound assay demonstrated that CPAE effectively reduce the migration speed of pancreatic cells in comparison to the untreated group (Figures 4A, B). Furthermore, Matrigel invasion assay demonstrated that non-cytotoxic CPAE was able to suppress pancreatic cancer cell invasion (Figure 4C). The above results collectively indicate that non-cytotoxic doses of CPAE inhibit pancreatic cancer cell migration and invasion.

### 3.5 CPAE reduces EMT in pancreatic cancer cells by suppressing the TGF-β1/smad pathway

**FIGURE 5:**
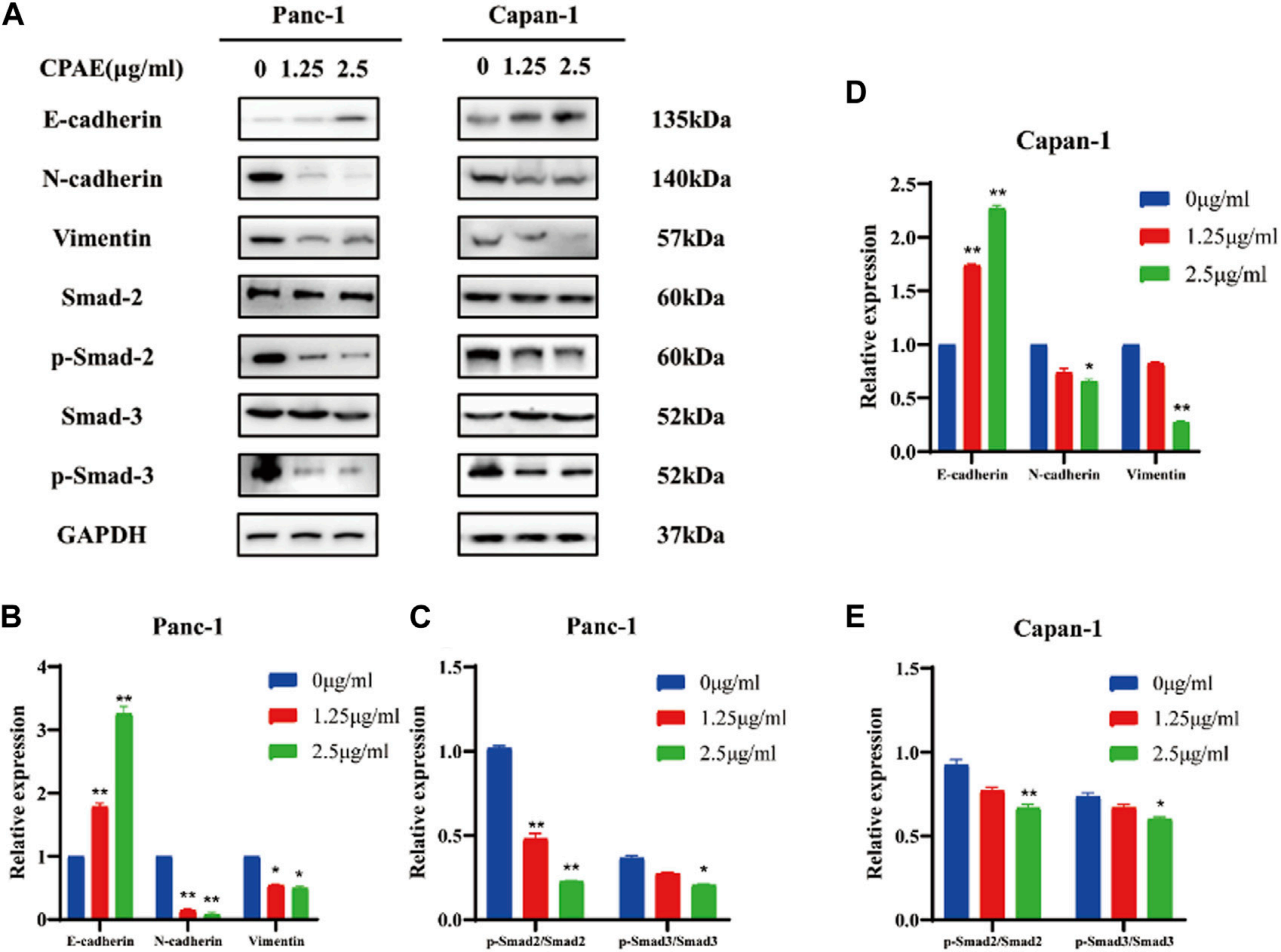
CPAE reduces the TGF-β1/Smad pathway, thereby inhibiting EMT. **(A)** Protein levels of E-cadherin, N-cadherin, Vimentin, Smad2, p-smad2, Smad3, and p-smad3 in cells after 1.25 or 2.5 μg/mL CPAE treatment for 48 h by immunoblotting assay **(B–E)** Histogram shows the level of proteins above in Panc-1 and Capan-1 cells. *p < 0.05, **p < 0.01.

The migration and invasion of pancreatic cancer is closely linked to EMT (Brabletz et al., 2021; Thiery, 2021). We observed that CPAE, at concentrations of 1.25 μg/mL and 2.5 μg/mL, increased E-cadherin expression while decreasing N-cadherin and vimentin expressions. These changes in cadherin and vimentin expression signify an alteration in the EMT process. Furthermore, we investigated the expression levels of EMT-related genes and found that after CPAE treatment, the levels of p-Smad2 and p-Smad3 decreased after CPAE treatment (Figures 5A, B). This observation suggests that CPAE reduces the TGF-β1/Smad pathway, ultimately inhibiting the process of EMT.

### 3.6 CPAE abrogates TGF-β1-induced metastasis in pancreatic cancer cells

**FIGURE 6:**
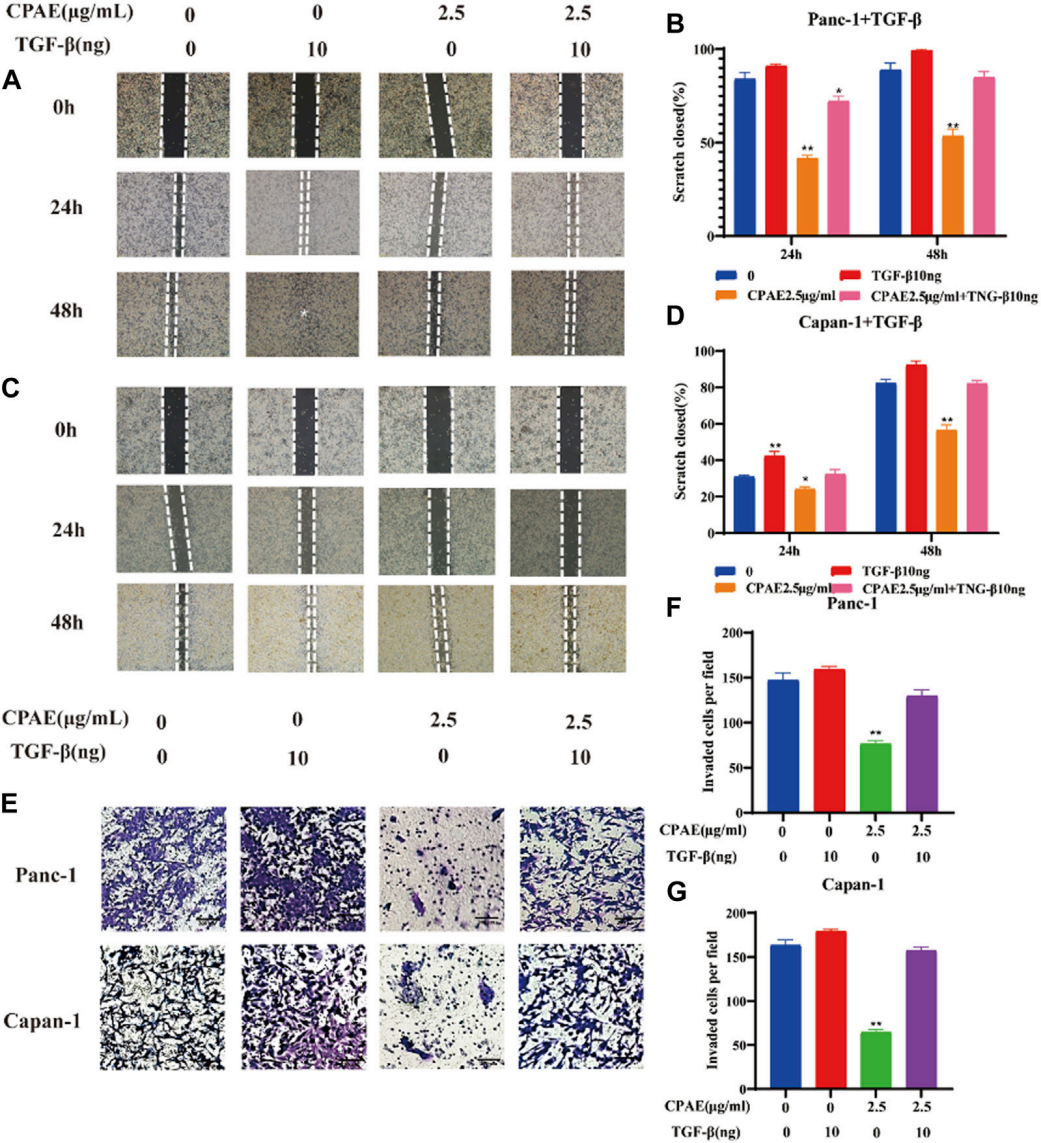
CPAE inhibits the metastatic effects induced by TGF-β1 in pancreatic cancer cells. **(A–D)** After treating cells with1.25 or 2.5 μg/mL CPAE and with or without TGF-β1, the cell migration is determined with a scratch wound-healing assay. The percentage of cell scratch closures after CPAE treatment was statistically compared. **(E)** Boyden Chamber assay demonstrated the effects of CPAE and TGF-β1 on the invasion of pancreatic cells. **(F,G)** The statistical comparison of invaded cells after CPAE treatment. *p < 0.05, **p < 0.01.

**FIGURE 7:**
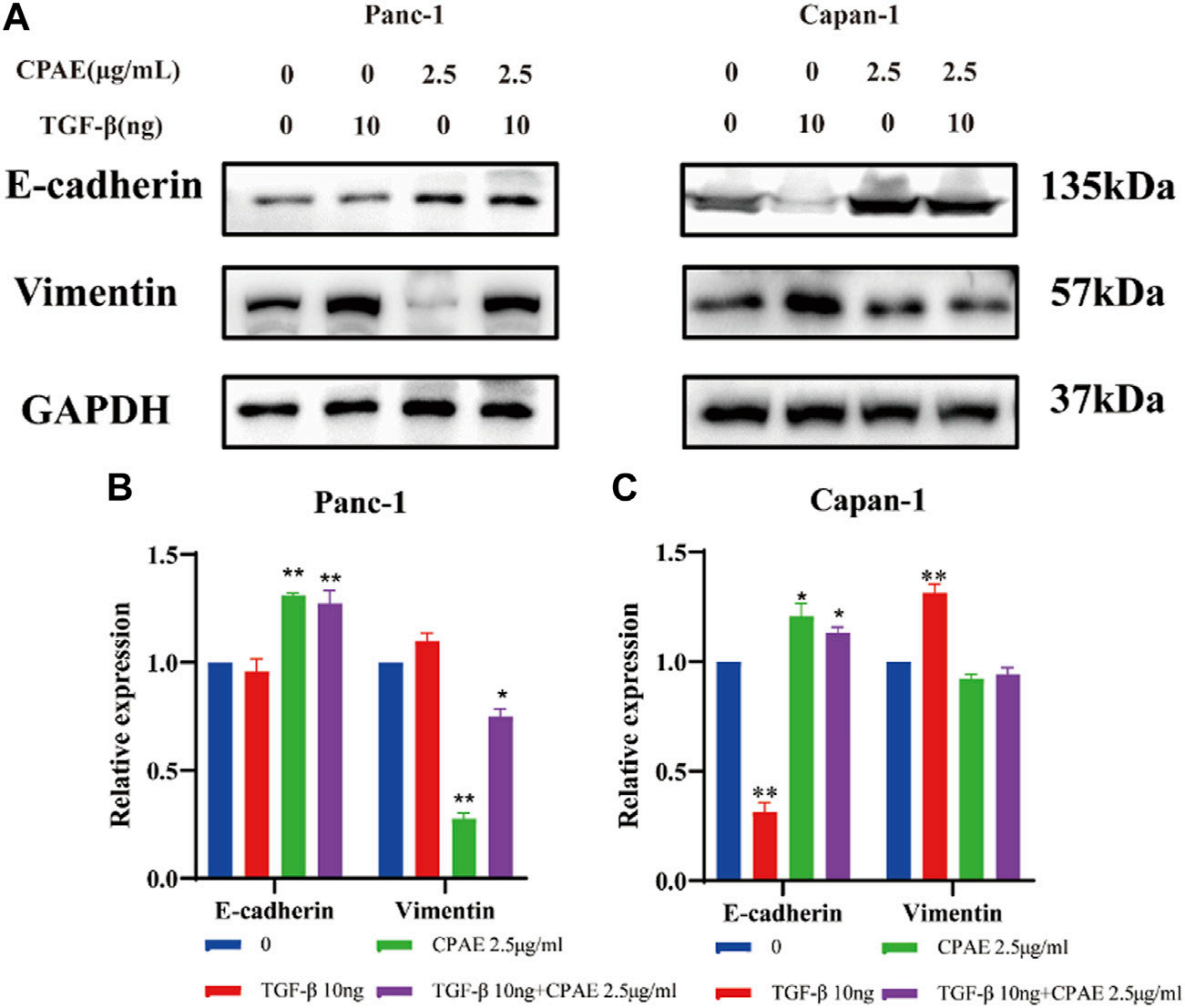
CPAE inhibits the EMT induced by TGF-β1. **(A)** Protein levels of E-cadherin and vimentin in cells after 2.5 μg/mL CPAE treatment and with or without TGF-β1 for 48 h by immunoblotting assay; **(B,C)** Histogram shows the levels of proteins above in pancreatic cancer cells. *p < 0.05, **p < 0.01.

To further investigate the inhibitory effects of CPAE on TGF-β1, we conducted experiments where cancer cells were exposed to TGF-β1 (10 ng/mL) with or without CPAE intervention. In the scratch-wound assay, it was shown that TGF-β1 increased the migration of pancreatic cancer cells, enhancing their metastatic potential. Conversely, the addition of CPAE effectively counteracted the TGF-β1-induced metastasis of pancreatic cancer cells (Figures 6A–D). Furthermore, the Matrigel invasion assay confirmed that TGF-β1 promoted cell invasion, while the presence of CPAE inhibited the invasion of cells induced by TGF-β1 (Figures 6E–G). As shown in Figure 7, the introduction of TGF-β1 induced EMT, as demonstrated by the decrease in E-cadherin and increase in vimentin protein expressions. These results collectively demonstrate that CPAE can mitigate the EMT induction caused by TGF-β1 and inhibit pancreatic cancer cell metastasis.

### 3.7 CPAE inhibits pancreatic tumor proliferation and metastasis *in vivo*

**FIGURE 8:**
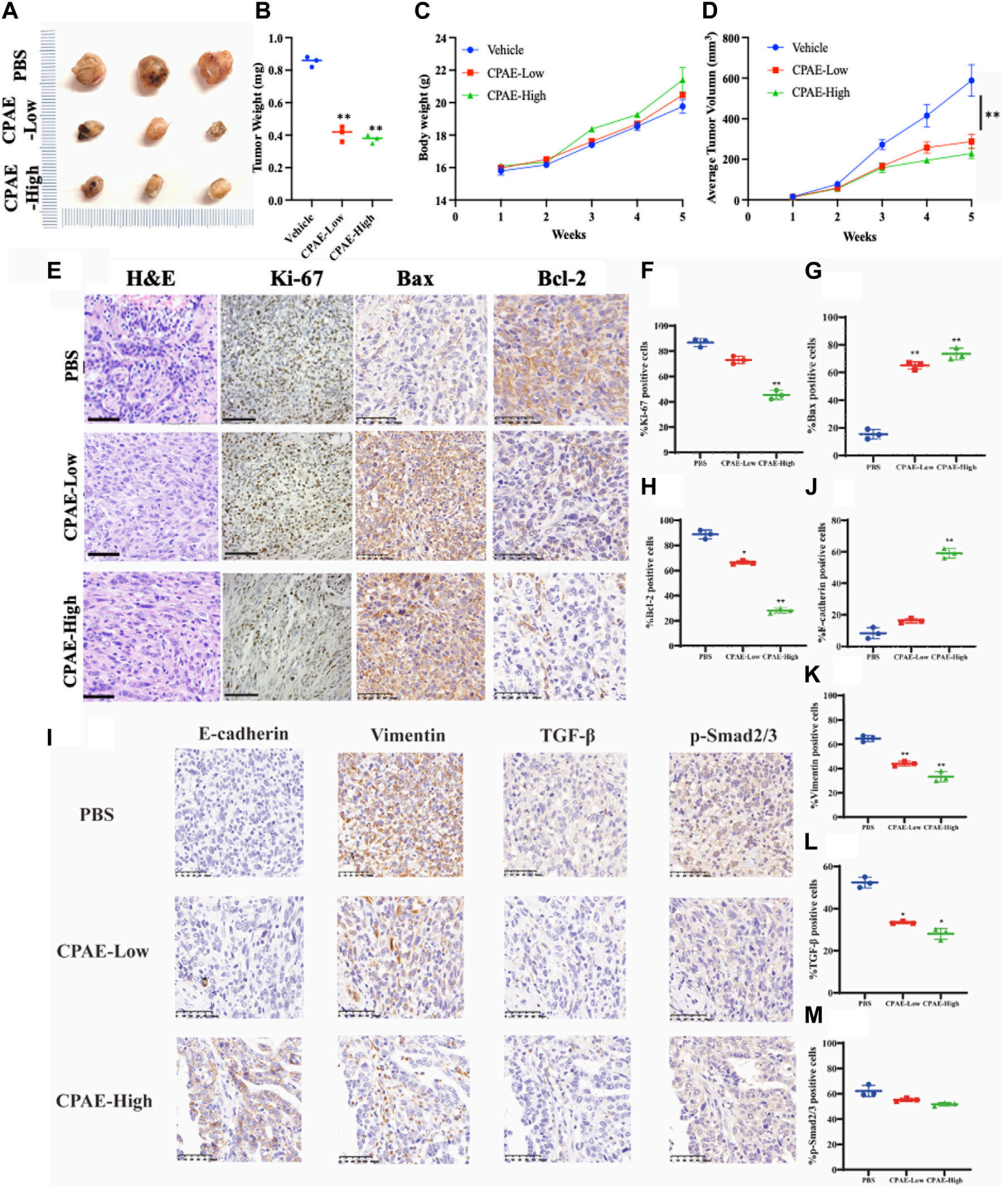
CPAE inhibits tumor proliferation and metastasis *in vivo*. **(A,B)** Mice treated with CPAE had a significant reduction in tumor size compared to that of the vehicle group treated with PBS by the end of treatment; **(C)** CPAE treatment showed minimal changes in mice body weights compared with the vehicle group and no significant difference in the weight of three groups was found; **(D)** 1 week after tumor inoculation, when tumors reached a volume of around 10 mm^3^, tumor-bearing mice were randomly divided into CPAE-low group (0.75 g/kg/2d, *n* = 3), CPAE-high group (1.5 g/kg/2d, *n* = 3), and vehicle control group (PBS, 0.2 mL/mice/time, *n* = 3) for receiving equivalent volume of designated treatment for 4 weeks, during which the tumor volume were measured and recorded. Data showed the average tumor volume in CPAE-low and -high groups were significantly smaller than that of the vehicle control group. **(E–H)** After CPAE treatment, the content of Bax increased, whereas the content of Bcl-2 decreased. **(I–M)** After CPAE treatment, the content of E-cadherin increased, whereas the content of vimentin and TGF-β1 decreased. **p < 0.01.

**FIGURE 9:**
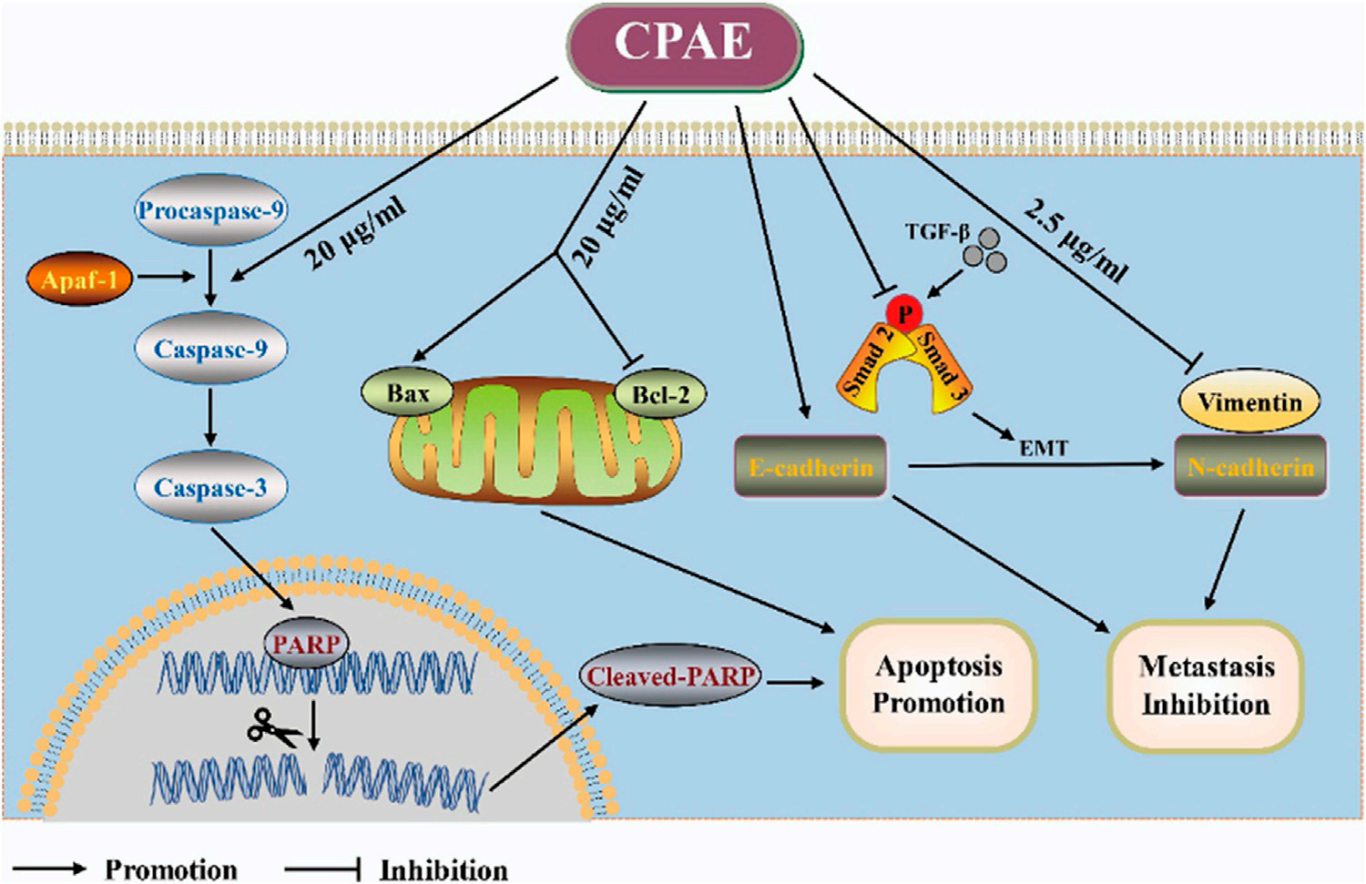
Schematic diagram. The anti-pancreatic cancer mechanism of CPAE by inducing apoptosis, inhibiting invasion and migration, and inducing the caspase-dependent PARP pathway and TGF-β-Smad2/3 signaling-mediated epithelial-mesenchymal transition.

In our pharmacological experiments, we followed the guidelines from a classic pharmacology textbook, which recommends converting equivalent doses based on the body surface area of humans and animals. As demonstrated in Figure 8, mice treated with CPAE exhibited a significant reduction in subcutaneous tumors compared to the control group. Remarkably, there were no significant differences in the weight of the three groups, and there were no discernible differences in the histological appearance between the treatment and control groups for heart, liver and kidney tissue (Supplementary Figure S2), indicating the CPAE treatment is safe at the selected concentration in murine model. Ki-67, a protein associated with nuclear division and cell proliferation, was notably reduced in the CPAE-treated group when compared to the control group. This reduction indicates that CPAE has the potential to inhibit tumor proliferation *in vivo*. After CPAE treatment, there was an increase in the levels of Bax, while the levels of Bcl-2 decreased. Simultaneously, there was an increase in the levels of E-cadherin, whereas the levels of vimentin and TGF-β1 decreased. However, there was no significant statistical difference in p-Smad2/3. These findings collectively suggest that CPAE effectively inhibits tumor proliferation and metastasis *in vivo.* In Figure 9, we summarize the anti-pancreatic cancer mechanism of CPAE, which involves inducing apoptosis, inhibiting invasion and migration, and regulating the caspase-dependent PARP pathway and TGF-β1-Smad2/3 signaling-mediated EMT.

## 4 Discussion

In the past decades, increasing research attention and progress have been made on anti-cancer natural product discovery (Wang et al., 2015; Kim and Kim, 2018; Liu et al., 2020). The well-known chemotherapy drug of the last 50 years, Paclitaxel, derived from the bark of the Pacific yew tree (*Taxus brevifolia*), is the most successful anti-cancer phytomedicine developed and has demonstrated a wide range of efficacy across various cancers (Zhang et al., 2017; Nazhand et al., 2020). Bufalin, one of the main ingredients of Bufonis venenum, has been recognized by the China Food and Drug Administration for its normal use in the treatment of various gastrointestinal cancers (Lan et al., 2019; Chen et al., 2021; Zou et al., 2021). Berberine hydrochloride, derived from *Coptis chinensis,* has demonstrated potential as an anti-cancer agent *in vitro* and *in vivo* and is currently under clinical trial for its preventive role for patients with colorectal cancer (Wang et al., 2017; Hesari et al., 2018; Imenshahidi and Hosseinzadeh, 2019; Rauf et al., 2021). Therefore, exploring the traditionally implicated herbs for cancer may provide a shortcut to novel drug discovery.

This study investigated the antitumor effects of CPAE *in vivo* and *in vitro*, including inhibiting proliferation, promoting apoptosis, and inhibiting invasion and migration. The MTT assay shows that with time and dose increase, CPAE significantly inhibits the proliferation of pancreatic cancer cells. Then the plate clones also showed that CPAE reduced colony formation of pancreatic cancer cells. Therefore, CPAE’s anti-proliferation was confirmed *in vitro*. The detection of apoptosis by flow cytometry showed that because of the increase of CPAE, the apoptosis ratio of pancreatic cancer cells was upregulated. Meanwhile, the cell cycle detection by flow cytometry indicated that CPAE could block pancreatic cancer cells to the G2 phase. At the protein level, it was further demonstrated that CPAE could increase the expression of Bax, reduce the level of Bcl-2, and activate the caspase-dependent PARP pathway, thereby promoting apoptosis.

Moreover, we also researched the effect of CPAE on metastasis in pancreatic cancer cells. Cell scratch assay and transwell chamber assay found that CPAE could reduce metastasis. Then the immunoblotting assay was used to demonstrate that CPAE upregulates E-ca levels while downregulating N-ca and vimentin expressions. We confirmed that CPAE plays a role in inhibiting EMT by inhibiting TGF-β1/Smad signaling. We also added TGF-β1 to further verify the influence of CPAE on pancreatic cancer cell metastasis. Finally, we constructed a subcutaneous tumor-bearing model of C57BL/6 J mice and detected the impact of CPAE *in vivo*. After CPAE treatment, the tumor volume of the mice decreased significantly. The amount of Ki67, E-cadherin, Smad, and TGF-β1 in the tumor was detected by H&E staining. These results indicate that CPAE inhibits tumor proliferation and metastasis *in vivo*.

Our current research significantly expands upon our previous work, which primarily focused on the single compound Paeonol derived from Cynanchum paniculatum and Paeonia suffruticosa roots. In this study, we shift our attention to the Cynanchum paniculatum aqueous extract (CPAE) as a potential anti-pancreatic cancer agent. This transition from a single compound to a complex herbal extract represents a notable advancement, allowing us to investigate the synergistic effects of multiple constituents within CPAE. Furthermore, our current study offers a more comprehensive mechanistic understanding of CPAE’s anti-cancer properties. While our previous work primarily targeted the inhibition of TGF-β1/Smad signaling to suppress migration and invasion in pancreatic cancer cells, our current research explores apoptosis induction through the Caspase-dependent PARP pathway and the inhibition of epithelial-mesenchymal transition (EMT) via the TGF-β1/Smad2/3 pathway. This broader mechanistic exploration enhances our insight into CPAE’s multifaceted anti-cancer effects. Additionally, we incorporate *in vivo* experiments using a murine xenograft model to validate CPAE’s anti-cancer effects. This represents a significant advancement compared to our prior focus on *in vitro* experiments and provides a clinically relevant context for evaluating CPAE’s potential as a pancreatic cancer therapeutic.

Adherence to rigorous quality control standards was pivotal in ensuring the consistency and reliability of *Xuchangqing*. The established guidelines stipulated a minimum paeonol content of 1.3%, as assessed by high-performance liquid chromatography (HPLC) fingerprint analysis of Radix *C. paniculatum* (China Medical and Technology Press, 2010). It is worth noting that variations in paeonol concentration within *C. paniculatum* can arise due to diverse cultivation conditions and extraction methodologies (China Medical and Technology Press, 2010; Yang and Wei, 2012). Additionally, other phenolic compounds, including chlorogenic acid, vanillic acid, hesperidin, paeonolide, and vincetoxicosid B have also been isolated and validated using HPLC as reported (Li and Li, 2022). Further comprehensive assessment of safety and quality control is warranted to investigate the responsible integration of Xuchangqing in the realm of cancer therapeutics. Moreover, it emphasizes the pivotal role of stringent quality control protocols when harnessing herbal extracts for prospective clinical applications.

The assessment of safety and quality of the experimental agent, Xuchangqing, employed in this study holds significant relevance within the broader context of its potential clinical applications. Previous investigations have undertaken comprehensive toxicity evaluations, revealing an LD50 value of 32.9 ± 1.0 g/kg upon intraperitoneal administration to mice. These findings indicate a favorable safety margin in murine models, a crucial consideration in translational research (Xi, 2006). Intravenous administration of Xuchangqing at a dose of 5 g/kg in rabbits elicited transient convulsions, followed by complete recovery within 48 h, underscoring a manageable acute toxicity profile (Xi, 2006). For the drug’s safety, we conducted a thorough examination of body weight and liver and kidney morphology of mice, and our findings indicated that CPAE is safe for mice without causing liver and kidney toxicity. CPAE, as a top-grade traditional Chinese medicine, is traditionally employed to invigorate the *qi,* a dynamic and holistic concept in TCM, encompassing both physical and energetic aspects of health and wellbeing. Modern pharmacology also confirms CPAE’s anti-inflammatory effects in various diseases. It is important to note that our study primarily focused on CPAE’s impact on tumor cells, and we acknowledge the need to consider its effects on the tumor microenvironment, inflammatory factors, and immune function. Particularly in *in vivo* experiments, the tumor microenvironment plays an integral role. Therefore, our future research endeavors will delve into studying the specific effects of CPAE on the microenvironment, including its interaction with fibroblasts. This will enable a more comprehensive exploration of the combined anti-inflammatory and antitumor mechanisms of CPAE.

The novelty of our current study lies in the investigation of CPAE, a complex herbal extract, as a promising anti-pancreatic cancer agent. This approach considers the holistic effects of numerous compounds present in CPAE, potentially offering a comprehensive and synergistic anti-cancer strategy. Furthermore, our research highlights CPAE’s dual mechanisms of action, targeting both apoptosis induction and EMT inhibition. This dual approach is relatively novel, as it simultaneously addresses cancer cell survival and metastatic potential, positioning CPAE as a promising and multifaceted anti-pancreatic cancer agent. However, further validation of the observed effects of CPAE, including on apoptotic pathways, TGF-β1/Smad signalling and EMT-related proteins using various methods, as well as comprehensively assess TGF-β expression in peripheral blood at various treatment time-points are warranted. It is worth mentioning that, in the TCM theory, CPAE is made up of many constituents. Specific components that mainly play an anti-tumor role still need to be further studied. In addition, large-scale clinical trials are needed in the future to observe the antitumor effect of CPAE on pancreatic cancer patients. Chinese medicines are often taken in the form of compound prescriptions, so the compatibility of CPAE is also worth exploring.

## 5 Conclusion

In summary, the clinical efficacy of CPAE in the treatment of pancreatic cancer is impressive, although the precise anti-tumor mechanism has been underexplored. Through our research, we have established that CPAE exhibits anti-proliferative, pro-apoptotic, anti-invasion, and anti-migration effects both *in vivo* and *in vitro.* We have identified that CPAE promotes apoptosis through the caspase-dependent PARP pathway and mitigates metastasis by inhibiting the TGF-β1/Smad pathway at non-cytotoxic doses. Our findings suggest that CPAE holds promise as a potential avenue for the future treatment of pancreatic cancer.

## Data Availability

The original contributions presented in the study are included in the article/Supplementary Material, further inquiries can be directed to the corresponding authors.

## Abbreviations

ATCC, American Type Culture Collection; CM, Chinese Medicine; CHM, Chinese herbal medicine; CPAE, *Cynanchum paniculatum* (Bunge) Kitag. ex H.Hara aqueous extract; DMEM, Dulbecco’s Modified Eagle’s Medium; DMSO, dimethyl sulfoxide; E-Ca, E-cadherin; EMT, epithelial-mesenchymal transition; FBS, fetal bovine serum; N-Ca, N-cadherin; PFA, paraformaldehyde; RIPA, Radioimmunoprecipitation assay buffer; TCM, Traditional Chinese medicine; TGF-β1, Transforming Growth Factor β1.

## References

[B1] Brabletz S. Schuhwerk H. Brabletz T. Stemmler M. P. (2021). Dynamic EMT: a multi-tool for tumor progression. EMBO J. 40 (18), e108647. 10.15252/embj.2021108647 34459003 PMC8441439

[B2] Cai J. Chen H. Lu M. Zhang Y. Lu B. You L. (2021). Advances in the epidemiology of pancreatic cancer: trends, risk factors, screening, and prognosis. Cancer Lett. 520, 1–11. 10.1016/j.canlet.2021.06.027 34216688

[B3] Chen J. Wang H. Jia L. He J. Li Y. Liu H. (2021). Bufalin targets the SRC-3/MIF pathway in chemoresistant cells to regulate M2 macrophage polarization in colorectal cancer. Cancer Lett. 513, 63–74. 10.1016/j.canlet.2021.05.008 34000344

[B4] Chen J. X. Cheng C. S. Chen J. Lv L. L. Chen Z. J. Chen C. (2020). Cynanchum paniculatum and its major active constituents for inflammatory-related diseases: a review of traditional use, multiple pathway modulations, and clinical applications. Evid. Based Complement. Altern. Med. 2020, 7259686. 10.1155/2020/7259686 PMC7396087 32774428

[B5] Cheng C. S. Chen J. X. Tang J. Geng Y. W. Zheng L. Lv L. L. (2020). Paeonol inhibits pancreatic cancer cell migration and invasion through the inhibition of TGF-β1/smad signaling and epithelial-mesenchymal-transition. Cancer Manag. Res. 12, 641–651. 10.2147/CMAR.S224416 32099461 PMC6996112

[B6] China Medical and Technology Press (2010) Pharmacopoeia of the people's Republic of China, China medical and Technology. Beijing, China: Press.

[B7] Chinese Pharmacopoeia Commission (2015) Chinese Pharmacopoeia commission Pharmacopoeia of the people's Republic of China. Beijing: China Medical Science Publisher.

[B8] Choudhary G. S. Al-Harbi S. Almasan A. (2015). Caspase-3 activation is a critical determinant of genotoxic stress-induced apoptosis. Methods Mol. Biol. 1219, 1–9. 10.1007/978-1-4939-1661-0_1 25308257

[B9] Chunhu Z. Suiyu H. Meiqun C. Guilin X. Yunhui L. (2008). Antiproliferative and apoptotic effects of paeonol on human hepatocellular carcinoma cells. Anticancer Drugs 19 (4), 401–409. 10.1097/CAD.0b013e3282f7f4eb 18454050

[B10] Collaborators G. B. D. P. C. (2019). The global, regional, and national burden of pancreatic cancer and its attributable risk factors in 195 countries and territories, 1990-2017: a systematic analysis for the Global Burden of Disease Study 2017. Lancet Gastroenterol. Hepatol. 4 (12), 934–947. 10.1016/S2468-1253(19)30347-4 31648972 PMC7026711

[B11] Colotta F. Allavena P. Sica A. Garlanda C. Mantovani A. (2009). Cancer-related inflammation, the seventh hallmark of cancer: links to genetic instability. Carcinogenesis 30 (7), 1073–1081. 10.1093/carcin/bgp127 19468060

[B12] Gao Y. Chen S. Sun J. Su S. Yang D. Xiang L. (2021). Traditional Chinese medicine may be further explored as candidate drugs for pancreatic cancer: a review. Phytother. Res. 35 (2), 603–628. 10.1002/ptr.6847 32965773

[B13] Hesari A. Ghasemi F. Cicero A. F. G. Mohajeri M. Rezaei O. Hayat S. M. G. (2018). Berberine: a potential adjunct for the treatment of gastrointestinal cancers? J. Cell Biochem. 119 (12), 9655–9663. 10.1002/jcb.27392 30125974

[B14] Imenshahidi M. Hosseinzadeh H. (2019). Berberine and barberry (Berberis vulgaris): a clinical review. Phytother. Res. 33 (3), 504–523. 10.1002/ptr.6252 30637820

[B15] Ji C.-Y. Tan S.-Y. Wang Y. (2007). Effects of bcl-2 and p53 in paeonol induced apoptosis of human colorectal cancer cell line HT-29 and the mechanism. Chin. General Pract. (05), 364–366. (Chinese).

[B16] Jiang J. Liu R. Zhang Z. Zhang X. Qi R. Chen S. (2019). Study on the treatment of pancreatic cancer with integrated traditional Chinese and Western medicine: a study protocol of a multicenter prospective cohort study. Med. Baltim. 98 (47), e17975. 10.1097/MD.0000000000017975 PMC6882619 31764804

[B17] Kim C. Kim B. (2018). Anti-cancer natural products and their bioactive compounds inducing er stress-mediated apoptosis: a review. Nutrients 10 (8), 1021. 10.3390/nu10081021 30081573 PMC6115829

[B18] Kim E. H. Min H. Y. Chung H. J. Song J. Park H. J. Kim S. (2012). Anti-proliferative activity and suppression of P-glycoprotein by (-)-antofine, a natural phenanthroindolizidine alkaloid, in paclitaxel-resistant human lung cancer cells. Food Chem. Toxicol. 50 (3-4), 1060–1065. 10.1016/j.fct.2011.11.008 22120505

[B19] Lan Y. L. Lou J. C. Jiang X. W. Wang X. Xing J. S. Li S. (2019). A research update on the anticancer effects of bufalin and its derivatives. Oncol. Lett. 17 (4), 3635–3640. 10.3892/ol.2019.10062 30915168 PMC6430489

[B20] Lee S. K. Nam K. A. Heo Y. H. (2003). Cytotoxic activity and G2/M cell cycle arrest mediated by antofine, a phenanthroindolizidine alkaloid isolated from Cynanchum paniculatum. Planta Med. 69 (1), 21–25. 10.1055/s-2003-37021 12567274

[B21] Li N. Fan L. L. Sun G. P. Wan X. A. Wang Z. G. Wu Q. (2010). Paeonol inhibits tumor growth in gastric cancer *in vitro* and *in vivo* . World J. Gastroenterol. 16 (35), 4483–4490. 10.3748/wjg.v16.i35.4483 20845518 PMC2941074

[B22] Li Z. Li Z. (2022). Simultaneous Determination of Six Phenolic Acids in the Extract of Cynanchi Paniculati Radix et Rhizoma by HPLC. China Pharm. 31 (21), 81–84. (Chinese). 10.3969/j.issn.1006-4931.2022.21.019

[B23] Liu T. (2016). Advances in the molecular biology mechanisms of xuchangqing’s anticancer effect. Pract. Med. J. 33 (5), 455–458. 10.14172/j.issn1671-4008,2016.05.032

[B24] Liu Y. Yang S. Wang K. Lu J. Bao X. Wang R. (2020). Cellular senescence and cancer: focusing on traditional Chinese medicine and natural products. Cell Prolif. 53 (10), e12894. 10.1111/cpr.12894 32881115 PMC7574878

[B25] Maisonneuve P. (2019). Epidemiology and burden of pancreatic cancer. Presse Med. 48 (3 Pt 2), e113–e123. 10.1016/j.lpm.2019.02.030 30878335

[B26] Min H. Y. Chung H. J. Kim E. H. Kim S. Park E. J. Lee S. K. (2010). Inhibition of cell growth and potentiation of tumor necrosis factor-α (TNF-α)-induced apoptosis by a phenanthroindolizidine alkaloid antofine in human colon cancer cells. Biochem. Pharmacol. 80 (9), 1356–1364. 10.1016/j.bcp.2010.07.026 20674553

[B27] Nazhand A. Durazzo A. Lucarini M. Mobilia M. A. Omri B. Santini A. (2020). Rewiring cellular metabolism for heterologous biosynthesis of Taxol. Nat. Prod. Res. 34 (1), 110–121. 10.1080/14786419.2019.1630122 31298589

[B28] Neoptolemos J. P. Kleeff J. Michl P. Costello E. Greenhalf W. Palmer D. H. (2018). Therapeutic developments in pancreatic cancer: current and future perspectives. Nat. Rev. Gastroenterol. Hepatol. 15 (6), 333–348. 10.1038/s41575-018-0005-x 29717230

[B29] Pan S. Y. (2017). “Clinical study of traditional Chinese medicine Aikang decoction in the treatment of cancer pain,” in The 5th world congress of integrative medicine abstacts collection (Guangzhou, Guangdong, China: Medicine Baltimore), 834.

[B30] Rauf A. Abu-Izneid T. Khalil A. A. Imran M. Shah Z. A. Emran T. B. (2021). Berberine as a potential anticancer agent: a comprehensive review. Molecules 26 (23), 7368. 10.3390/molecules26237368 34885950 PMC8658774

[B31] Siegel R. L. Miller K. D. Jemal A. (2019). Cancer statistics, 2019. CA Cancer J. Clin. 69 (1), 7–34. 10.3322/caac.21551 30620402

[B32] Slade D. (2020). PARP and PARG inhibitors in cancer treatment. Genes Dev. 34 (5-6), 360–394. 10.1101/gad.334516.119 32029455 PMC7050487

[B33] Tang J. Feng Y. Tsao S. Wang N. Curtain R. Wang Y. (2009). Berberine and Coptidis rhizoma as novel antineoplastic agents: a review of traditional use and biomedical investigations. J. Ethnopharmacol. 126 (1), 5–17. 10.1016/j.jep.2009.08.009 19686830

[B34] Thiery J. P. (2021). EMT: an update. Methods Mol. Biol. 2179, 35–39. 10.1007/978-1-0716-0779-4_6 32939712

[B35] Wang K. Feng X. Chai L. Cao S. Qiu F. (2017). The metabolism of berberine and its contribution to the pharmacological effects. Drug Metab. Rev. 49 (2), 139–157. 10.1080/03602532.2017.1306544 28290706

[B36] Wang N. Tan H. Y. Li L. Yuen M. F. Feng Y. (2015). Berberine and Coptidis Rhizoma as potential anticancer agents: recent updates and future perspectives. J. Ethnopharmacol. 176, 35–48. 10.1016/j.jep.2015.10.028 26494507

[B37] Wang Y. Luo W. Wang Y. (2019). PARP-1 and its associated nucleases in DNA damage response. DNA Repair (Amst) 81, 102651. 10.1016/j.dnarep.2019.102651 31302005 PMC6764844

[B38] Weon J. B. Lee B. Yun B. R. Lee J. Ma C. J. (2012). Simultaneous determination of ten bioactive compounds from the roots of Cynanchum paniculatum by using high performance liquid chromatography coupled-diode array detector. Pharmacogn. Mag. 8 (31), 231–236. 10.4103/0973-1296.99289 23060698 PMC3466459

[B39] Wu Y. Cheng C. S. Li Q. Chen J. X. Lv L. L. Xu J. Y. (2021). The application of citrus folium in breast cancer and the mechanism of its main component nobiletin: a systematic review. Evid. Based Complement. Altern. Med. 2021, 2847466. 10.1155/2021/2847466 PMC8260297 34257674

[B40] Xi X. (2006) Analytical chemistry, China traditional Chinese medicine. Beijing, China: Publishing House.

[B41] Yang C. L. Wei Y. (2012). Fluorimetric analysis of paeonol in Chinese herbal medicine Cynanchi Paniculati Radix by aluminum ion-sensitized fluorescence. Acta Pharm. Sin. B 2 (3), 294–299. 10.1016/j.apsb.2012.01.008

[B42] Yang X. Hao J. Zhu C. H. Niu Y. Y. Ding X. L. Liu C. (2015). Survival benefits of western and traditional Chinese medicine treatment for patients with pancreatic cancer. Med. Baltim. 94 (26), e1008. 10.1097/MD.0000000000001008 PMC4504629 26131801

[B43] Zhang L. Tao L. Shi T. Zhang F. Sheng X. Cao Y. (2015). Paeonol inhibits B16F10 melanoma metastasis *in vitro* and *in vivo* via disrupting proinflammatory cytokines-mediated NF-κB and STAT3 pathways. IUBMB Life 67 (10), 778–788. 10.1002/iub.1435 26452780

[B44] Zhang M. Chen Y. Nie L. Jin X. Fu C. Yu L. (2017). Molecular, structural, and phylogenetic analyses of Taxus chinensis JAZs. Gene 620, 66–74. 10.1016/j.gene.2017.04.005 28390989

[B45] Zheng Y. (2008). Effects of Xu changqing decoction on serum cytokines in patients with acute pancreatitis. Traditional Chin. Med. Res. (05), 31–32.

[B46] Zheng Y. Gao F. Y. (2008). Clinical observation of Xu Changqing decoction in the treatment of patients with acute pancreatitis. Traditional Chin. Med. J. (05), 45–47.

[B47] Zhou X. Xia W. Zhang Y. Ma J. Zhou H. Dong L. (2020). Cynanchum paniculatum (Bunge) Kitag. ex H. Hara: a review of its ethnopharmacology, phytochemistry and pharmacology. J. Ethnopharmacol. 260, 112994. 10.1016/j.jep.2020.112994 32473366

[B48] Zou D. Song J. Deng M. Ma Y. Yang C. Liu J. (2021). Bufalin inhibits peritoneal dissemination of gastric cancer through endothelial nitric oxide synthase-mitogen-activated protein kinases signaling pathway. FASEB J. 35 (5), e21601. 10.1096/fj.202002780R 33913201

