# Supplementary figures and images for: Cynanchum paniculatum (Bunge) Kitag. ex H.Hara inhibits pancreatic cancer progression by inducing caspase-dependent apoptosis and suppressing TGF-β-mediated epithelial-mesenchymal transition
Source: Front Pharmacol. 2024 May 31;15:1284371. doi: 10.3389/fphar.2024.1284371 (PMC11176445; doi:10.3389/fphar.2024.1284371)

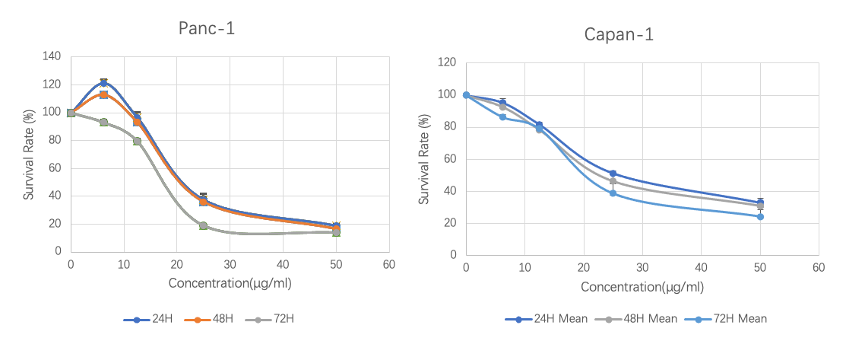

Supplement: Supplementary file 1 [file DataSheet1.zip › Supplementary Figure S1.tiff]

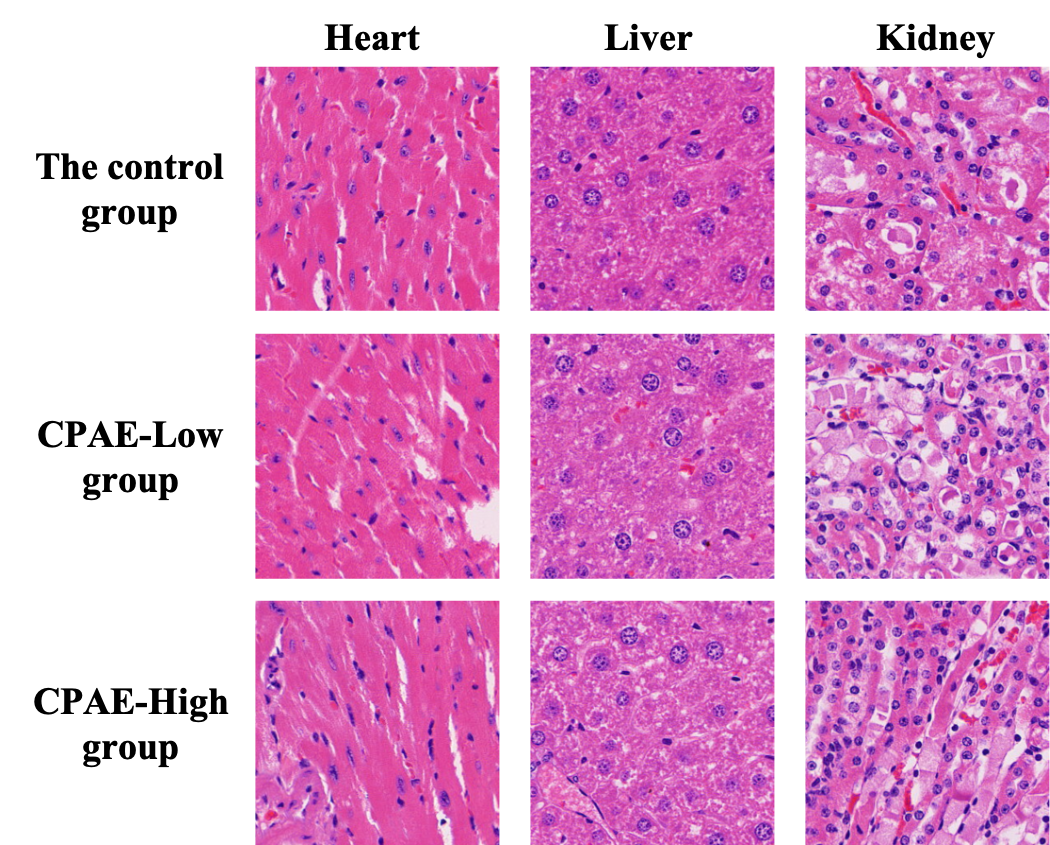

Supplement: Supplementary file 1 [file DataSheet1.zip › Supplementary Figure S2.tiff]
